# Supplementary figures and images for: Multiple Pathways to Homothallism in Closely Related Yeast Lineages in the Basidiomycota
Source: mBio. 2021 Feb 16;12(1):e03130-20. doi: 10.1128/mBio.03130-20 (PMC8545103; doi:10.1128/mBio.03130-20)

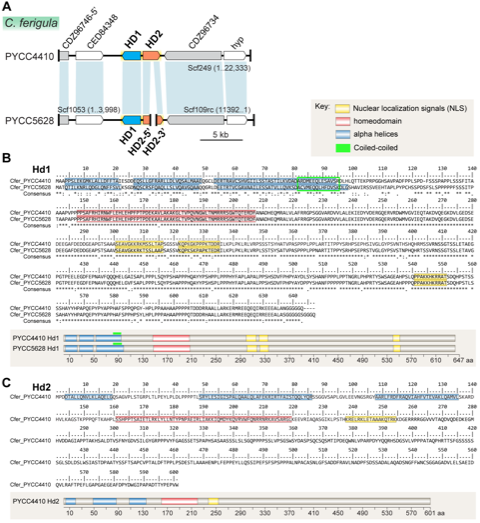

Supplement: FIG S1 [file mbio.03130-20-s0001.tif]

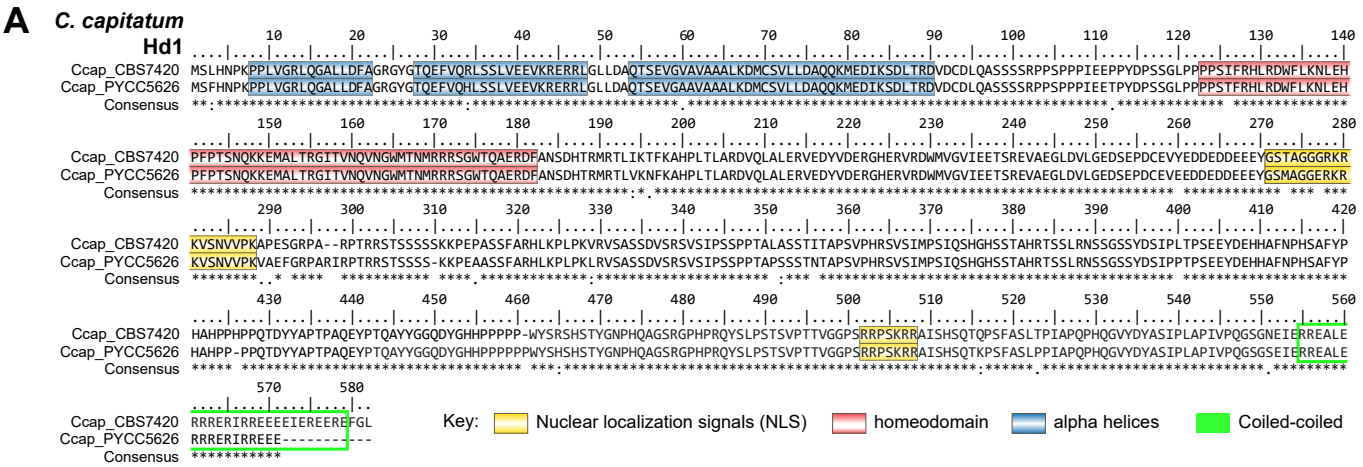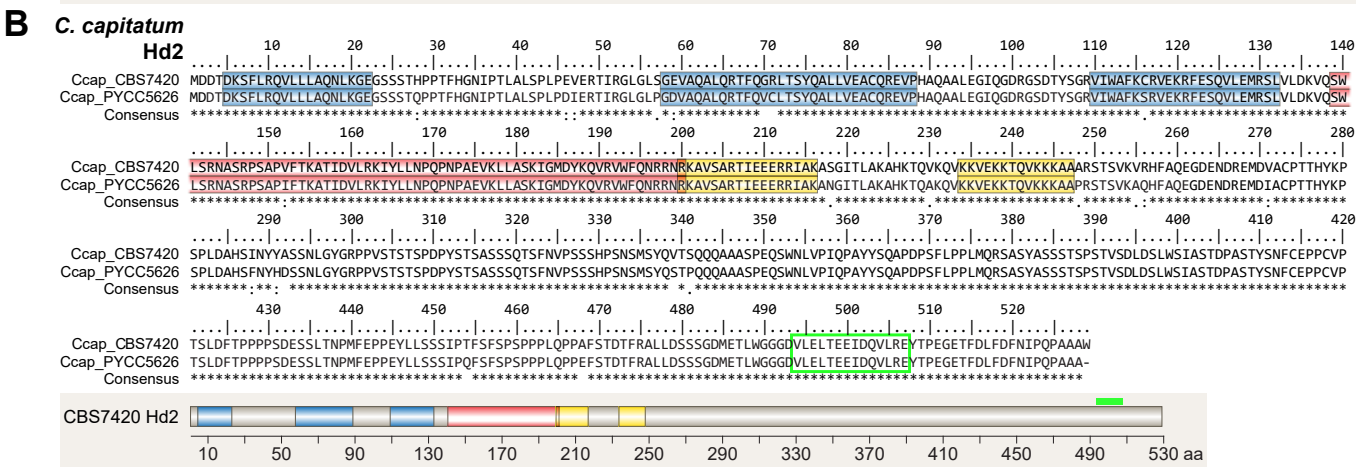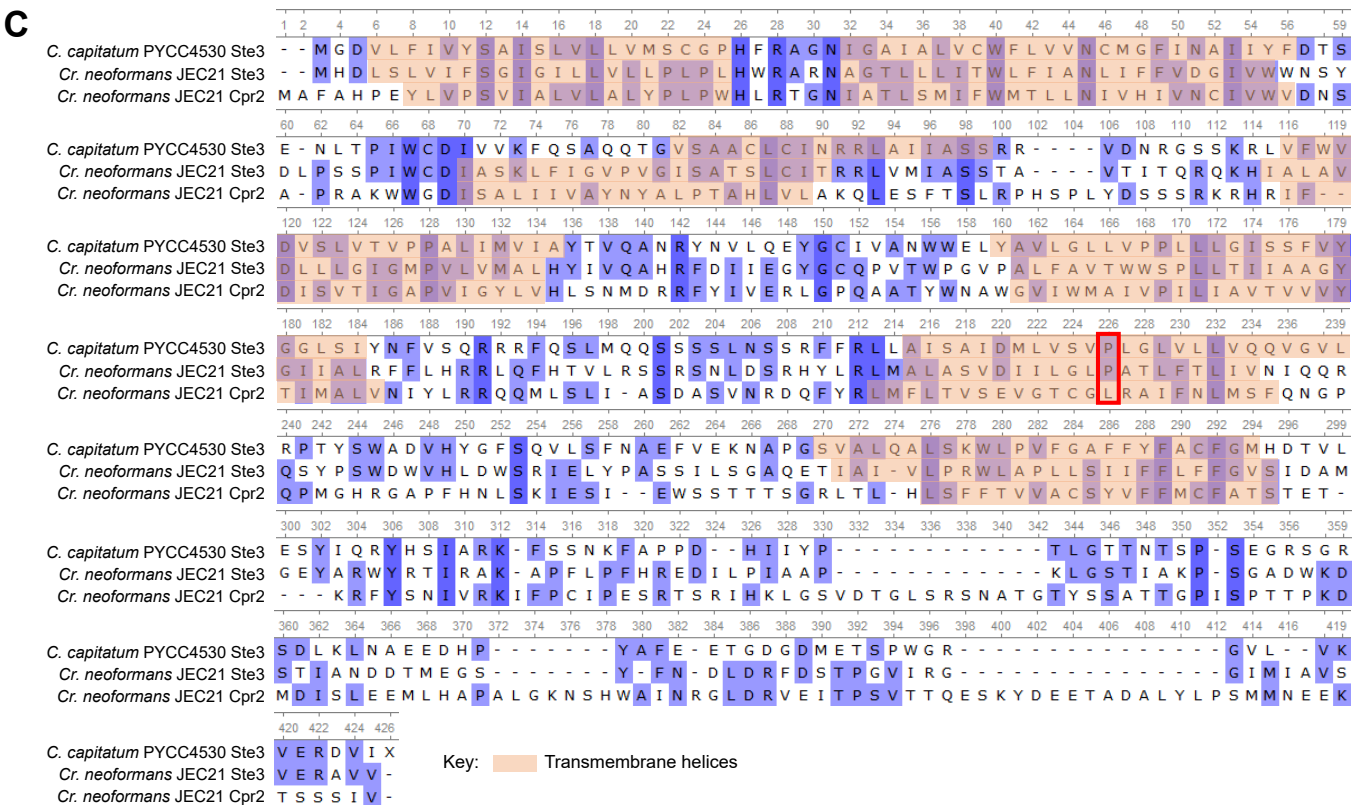

Supplement: FIG S2 [file mbio.03130-20-s0002.pdf]

**A***C. capitatum* PYCC4530

Scf 12

71.5 72 72.5 73 73.5 74 74.5 75 75.5 kb

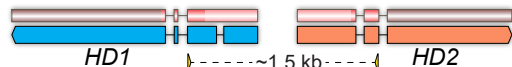*C. ferigula* PYCC4410

Scf 249

7.5 8 8.5 9 9.5 10 10.5 11 11.5 kb

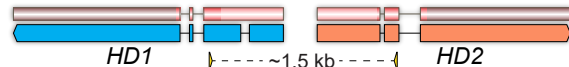**B**5' end of  
*HD1*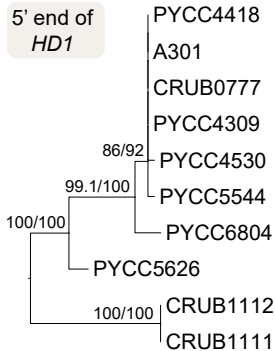5' end of  
*HD2*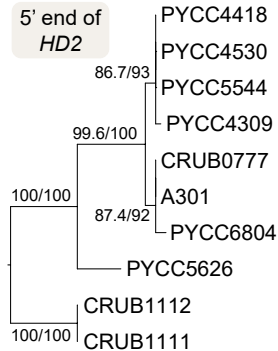**C**5' end of  
*HD1*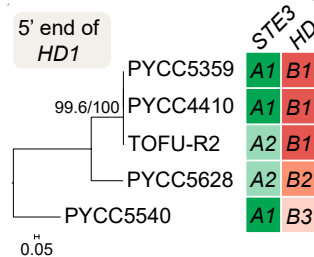

Supplement: FIG S3 [file mbio.03130-20-s0003.pdf]

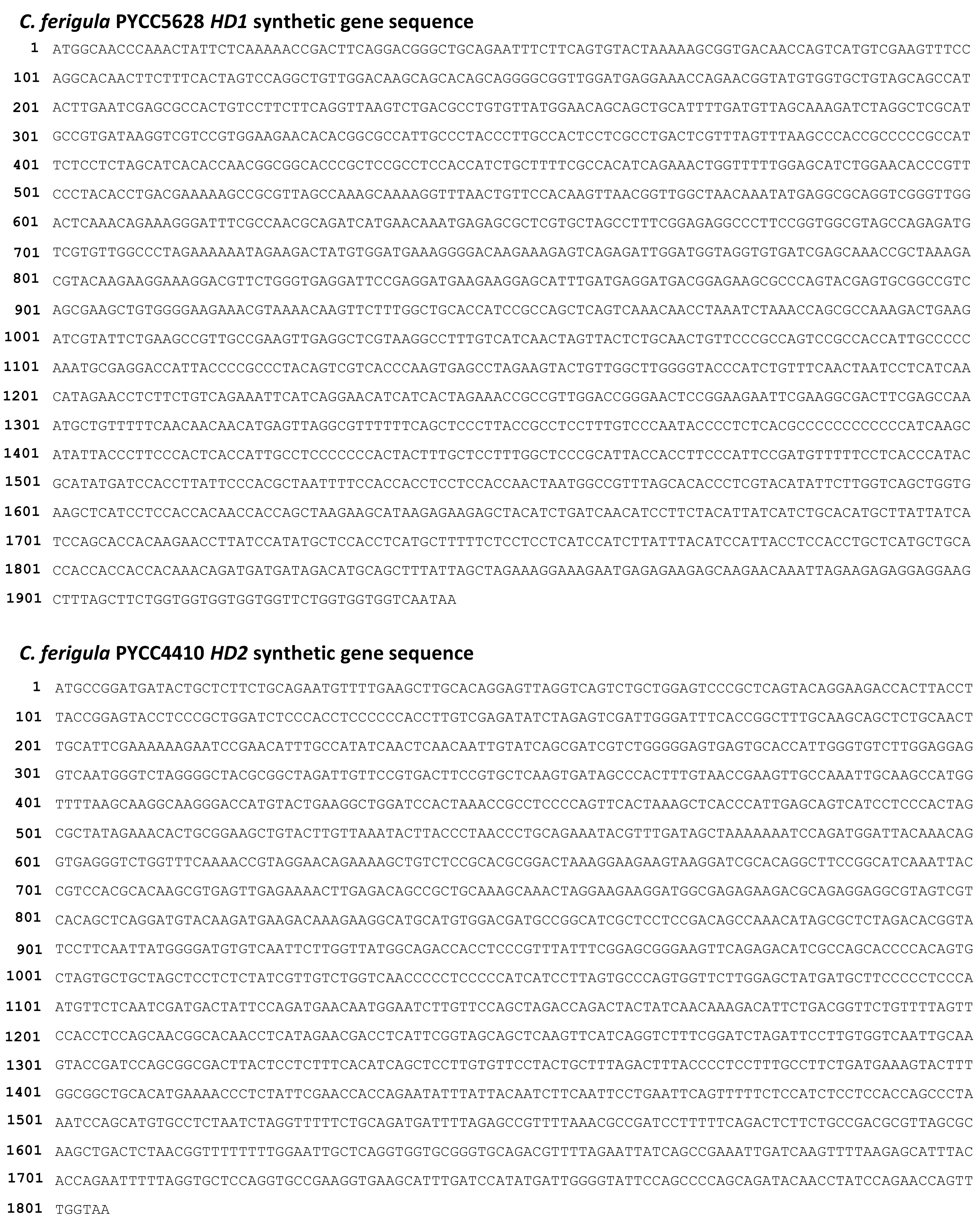

Supplement: FIG S4 [file mbio.03130-20-s0004.tif]

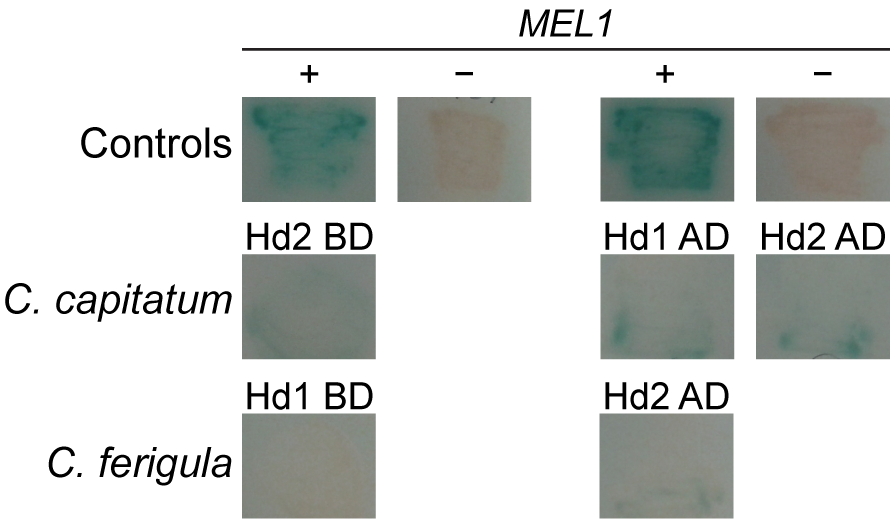

Supplement: FIG S5 [file mbio.03130-20-s0005.tif]
